# Impact of environmental microbiota on human microbiota of workers in academic mouse research facilities: An observational study

**DOI:** 10.1371/journal.pone.0180969

**Published:** 2017-07-13

**Authors:** Peggy S. Lai, Joseph G. Allen, Diane S. Hutchinson, Nadim J. Ajami, Joseph F. Petrosino, Thomas Winters, Christopher Hug, Gary R. Wartenberg, Jose Vallarino, David C. Christiani

**Affiliations:** 1 Division of Pulmonary and Critical Care, Massachusetts General Hospital, Boston, MA, United States of America; 2 Department of Environmental Health, Harvard T.H. Chan School of Public Health, Boston, MA, United States of America; 3 Harvard Medical School, Boston, MA, United States of America; 4 Alkek Center for Metagenomics and Microbiome Research, Department of Molecular Virology and Microbiology, Baylor College of Medicine, Houston, TX, United States of America; 5 Division of Pulmonary and Respiratory Diseases, Boston Children’s Hospital, Boston, MA, United States of America; Forschungszentrum Borstel Leibniz-Zentrum fur Medizin und Biowissenschaften, GERMANY

## Abstract

**Objectives:**

To characterize the microbial environment of workers in academic mouse research facilities using endotoxin, 16S qPCR, and 16S amplicon sequencing. To determine whether the work microbiome contributes to the human microbiome of workers.

**Methods:**

We performed area air sampling from the animal rooms, dirty, middle, and setup cage wash locations in four academic mouse research facilities. 10 workers in the dirty cage wash area underwent personal air sampling as well as repeated collection of nasal, oral, and skin samples before and after the work shift. Environmental samples underwent measurement of endotoxin, mouse allergen, bacteria copy number via 16S qPCR, and microbial identification via 16S rDNA sequencing. 16S rDNA sequencing was also performed on human samples before and after the work shift. SourceTracker was used to identify the contribution of the work microbiome to the human microbiome.

**Results:**

Median endotoxin levels ranged from undetectable to 1.0 EU/m^3^. Significant differences in mouse allergen levels, bacterial copy number, microbial richness, and microbial community structure were identified between animal, dirty, middle, and setup cage wash locations. Endotoxin levels had only a moderate correlation with microbial composition. Location within a facility was a stronger predictor of microbial community composition (R^2^ = 0.41, p = 0.002) than facility. The contribution of the work microbiome to the pre-shift human microbiome of workers was estimated to be 0.1 ± 0.1% for the oral microbiome; 3.1 ± 1.9% for the nasal microbiome; and 3.0 ± 1.5% for the skin microbiome.

**Conclusions:**

The microbial environment of academic animal care facilities varies significantly by location rather than facility. Endotoxin is not a proxy for assessment of environmental microbial exposures using 16S qPCR or 16S rDNA sequencing. The work microbiome contributes to the composition of the nasal and skin microbiome of workers; the clinical implications of this observation should be further studied.

## Introduction

High level microbial exposures are common in many work settings and linked to health effects [1]. Historically, microbial toxins such as endotoxin has been used as the measure of environmental microbes in health-related studies, with exposure limits in some countries based on these metrics [2]. However, epidemiologic studies on the relationship between endotoxin exposure and respiratory health have reported conflicting associations, with one recent analysis of three large European asthma birth cohorts finding a protective effect of endotoxin exposure in the Spanish cohort, no effect of endotoxin exposure in the German cohort, and a harmful effect of endotoxin exposure in the Dutch cohort despite similar approaches to environmental assessment and outcome definition [3]. These results raise the question of whether there are alternate ways to measure environmental microbial exposure that may better explain the observed variation in health effects.

Newer molecular methods to measure microbes have emerged based on the observation that the 16S ribosomal RNA (rRNA) gene is present in every bacteria, yet has distinct polymorphisms that can serve as a “fingerprint” for bacterial identity [4]. In recent years, due to the advent of next-generation sequencing and large databases linking the 16S ribosomal rRNA gene sequences (16S rDNA) to bacterial identity, it is now possible to rapidly identify the community of microbes present in a sample. Studies on the environmental microbiome mostly relate to pediatric studies, and have identified a link between environmental microbial exposure measured in this way and asthma. These studies have suggested that early life exposure to a high diversity of microbes is protective against atopy and asthma development [5], but conversely, in established asthma, exposure to a high diversity of microbes is associated with increased asthma severity [6]. Simultaneously, there is a growing recognition that the human microbiome is associated with asthma, with studies suggesting that the composition of the gut microbiome in early life predicts asthma development [7]. Other studies have examined the airway microbiome, and found differences in sputum microbial composition between asthmatics and non-asthmatics [8], and further identified associations between the airway microbiome and corticosteroid responsiveness in asthma [9].

Few studies, however, have examined the influence of the environmental microbiome on the human microbiome in adults. This may be related to observations that the human microbiome undergoes rapid changes only early in life during a time of rapid colonization [10]. after which it appears largely stable [11]. Yet, a few observational studies contradict the idea that the adult human microbiome cannot be perturbed by environmental microbial exposures. These studies largely revolve around animal exposures. A wide variety of animals are known to be a reservoir for methicillin resistant *Staphylococcus aureus* (MRSA). MRSA colonization is a known occupational hazard for veterinarians and technicians with animal contact compared to control subjects without animal contact [12]. An intriguing study in an agricultural region of North Carolina found that subjects living in census blocks with higher densities of swine also had higher rates of MRSA colonization in their nares; none of the subjects were livestock workers, precluding direct occupational exposures as the explanation [13]. A recent study of poultry abattoir workers found substantial differences in the gut microbiome of workers over a five month period [14]. It is clear that acquisition of bacteria from animal-related work is a potential occupational hazard.

We chose to study the effect of environmental microbial exposures on the human microbiome in animal care workers. Up to 125,000 workers are exposed to laboratory animals as part of their occupation in the United States [15]. A quarter of workers report upper respiratory symptoms due to laboratory animal exposure [16, 17], with a subset developing occupational asthma [18]. While any exposure (even indirect) to laboratory animals is associated with the development of symptoms [19]. workers involved in the direct care of animals including washing dirty cages have been shown to be at the highest risk for developing laboratory animal related allergy or asthma, likely due to the magnitude of their workplace exposures [20].

The purpose of this study was first to determine whether traditional methods of measuring microbial exposure using endotoxin can serve as a reasonable surrogate for sequencing-based measures of microbial exposure; historically, the vast majority of occupational studies rely on endotoxin measurement, and it is important to understand whether future studies relying on sequencing of microbial DNA can be extrapolated to settings with high endotoxin levels. Secondarily, our goal was to determine whether the human microbiome of animal care workers is impacted by the microbes in their work environment. We hypothesized that 1) measures of airborne endotoxin (found only in gram-negative bacteria) is not a surrogate for bacterial copy number (measured using real-time polymerase chain reaction of the 16S rRNA gene, or qPCR), nor is it a surrogate for bacterial identity and community structure (measured with amplicon sequencing of the 16S rRNA gene); 2) Similar to the human microbiome, locations with specific functions in each animal care facility (analogous to a body site) is a stronger predictor of microbial community structure than facility identity (analogous to an individual person); 3) A proportion of the human microbiome in animal care workers can be traced to their work microbiome.

## Materials and methods

### Environmental sample collection

Four academic research mouse facilities participated in this study. For our first aim, we sampled four locations with distinct functions within each facility; a representative animal room as well as the dirty, middle, and setup portions of the cage wash area (see **S1 Fig**). Animal rooms are locations where the mice are housed, typically 4 mice per cage with 3 of the 4 facilities having ventilated cages. Within the cage wash areas, the dirty location is where the contents of used cages (including mouse fecal matter) are dumped, and cages subsequently placed in an industrial washer. In the middle location, washed cages are set in racks to dry. In the setup location, dried cages are filled with wood chip litter (Alpha Chip, Northeastern Products, Warrensburg, New York) and food (Pico Lab Mouse 5058 High Fat Chow or Pico Rodent 5053 Maintenance Chow, St. Louis, Missouri) before autoclaving.

Area sampling was performed in each location of each facility. Paired irradiated sterile endotoxin-free 0.45 μm polycarbonate filters in closed faced cassettes (Zefon, Ocala, FL) were attached to area samplers (BGI, Butler, NJ) at a pump flow rate of 2.5 liters per minute and placed within one meter of workers for 8 hours during standard work hours. Paired field blanks were collected, consisting of sterile filters brought to the field site but not attached to a sampling pump, and subsequently underwent the same downstream analysis procedures.

### Human sample collection

For our second aim, we recruited 10 workers working in the dirty cage wash area of the four facilities. Inclusion criterion was current work in a dirty cage wash area of the involved animal care facilities. Exclusion criterion was use of antibiotics, corticosteroids, or immune-suppressing medications in the prior six months. Informed consent was obtained from all participants and the protocol was approved by the Institutional Review Board of Boston Children’s Hospital (protocol # IRB-P00015250). Prior to the start of their work shift, we obtained the following samples: nasal samples by rubbing sterile nylon flocked swabs (Puritan, Guilford. ME) in each anterior nare, oral wash by having workers swish 5 mL of sterile saline in their mouths then spit into a sterile specimen cup, and skin samples by rubbing sterile nylon flocked swabs (Puritan, Guilford. ME) in the retro-auricular crease for 30 seconds. These procedures were repeated immediately after the work shift. Exhaled nitric oxide and pre-bronchodilator spirometry was also performed at this time. Personal air samples were obtained from each participant as follows: Paired irradiated sterile endotoxin-free 0.45 μm polycarbonate filters in closed faced cassettes (Zefon, Ocala, FL) were attached to personal sampling pumps (Casella, Buffalo, NY) at a pump flow rate of 2.5 liters per minute. These samplers were worn by each participant for the duration of their 8-hour work shift.

All filters and biologic specimens were immediately placed on dry ice after sample collection, then stored at -80 degrees Celsius until subsequent processing and analysis in a single batch.

### Sample processing

For each pair of filters, one filter was eluted with pyrogen-free water and 0.05% TWEEN-20 for endotoxin analysis using the Limulus Amebocyte Lysate assay (Bio-Whittaker, Walkersville, MD), and mouse allergen analysis (Mus m 1) using the MARIA assay (Indoor Biotechnologies, Charlottesville, VA). The other filter, as well as all biological specimens, underwent microbial DNA extraction. Bacterial genomic DNA extraction methods used were adapted from the methods developed for the NIH-Human Microbiome Project [21]. Briefly, bacterial genomic DNA was extracted using MO BIO PowerSoil DNA Isolation Kit (MO BIO, Carlsbad, CA) following the manufacturer’s instructions. One sixth of each filter was used due to the small size of the beadbeating tubes. Extracted DNA concentrations were measured by Qubit (Life Technologies, Carlsbad, CA) for subsequent normalization of quantitative PCR results.

### Quantitative PCR (qPCR)

qPCR on extracted DNA from area air samples was performed in a QuantStudio 7 Flex Real-Time PCR System using MicroAmp Fast Optical 96-well and 384-well reaction plates (0.1ml), MicroAmp optical adhesive film, (all Applied Biosystems) and PerfeCTa SYBR Green FastMix, Low Rox (Quanta Biosciences). Each reaction contained 10ul of 2X Master Mix, 4 ul of DNA template, 625 nM of each primer (IDT), and PCR grade water to a final volume of 20 ul. Amplification was comprised of a 10-minute activation step at 95°C, followed by 40 cycles of 95°C for 10 s, 60°C for 30 s, and a fluorescence measurement. Melting curve analysis was done by monitoring fluorescence throughout incremental increases of temperature from 60°C to 95°C.

The qPCR primers (1369F-1492R) [22] target regions flanking V9 of the 16S rRNA gene. The standard curve was made using a serially diluted plasmid that contains nt 1369 to 1492 of an *E*. *coli* 16S rRNA gene. The concentrations of unknowns are calculated from C_T_ values using the equation generated from plotting the standard curve. All samples are run in triplicate, including the standard curve, a set of non-template controls (NTC), and inhibitor controls (known positives + unknown DNA).

### 16S amplicon sequencing

The 16S rDNA V4 region was amplified by polymerase chain reaction and sequenced on the Illumina MiSeq platform using the 2x250 bp paired-end protocol yielding paired-end reads that overlap almost completely. The primers used for amplification [23] contain adapters for MiSeq sequencing and single-end barcodes allowing pooling and direct sequencing of PCR products. Positive and negative controls were included in the run, including a field blank from air sampling. Raw sequence data was deposited in the NCBI read sequence archive (see **S1 Table** for accession numbers).

### Bioinformatics processing and statistical analysis

Endotoxin, 16S rDNA copy number, and mouse allergen quantities from each filter were divided by the total volume of air sampled to obtain concentrations in endotoxin units per cubic meter (EU/m^3^), copy numbers per cubic meter (copies/m^3^), or nanograms per cubic meter (ng/m^3^), respectively. Summary statistics were calculated using median [interquartile range] given the skewed distribution of the measurements, and the Kruskal-Wallis rank sum test was used to test for differences in each exposure metric between locations and facilities.

For sequencing data, reads were merged using USEARCH v7.0.1090 with no mismatches allowed and a minimum 50 bp overlap between reads. An expected error filter of 0.05 was applied. Reads were iteratively clustered to 97% similarity using UPARSE [24] and *de novo* chimera removal was performed. UCHIME [25] was applied for reference based chimera removal. All remaining reads were mapped to the SILVA database (version 123) [26] to determine taxonomy. Any reads that did not map to bacteria were excluded from subsequent analysis.

The resulting operational taxonomic unit (OTU) table, taxonomy table, and covariate table were merged using *phyloseq* [27] for downstream analysis including data transformation and calculation of diversity indices. In order to filter out potentially spurious taxa due to sequencing error, only OTUs present in at least 5% of samples (e.g. based on our sample size, OTUs present in at least 5 samples) were retained. Measures of alpha diversity were calculated including observed OTUs, Chao 1, Shannon, and inverse Simpson indices. A sensitivity analysis was performed on OTU tables rarefied to an equal sequencing depth of 1000 reads per sample to determine whether uneven sequencing depth affected diversity measures. To test the hypothesis that measures of alpha diversity differed across location, linear models with location and facility were used as predictors.

Given concerns that rarefaction is considered statistically inadmissible [28], we pursued an alternate workflow to address uneven sequencing depth across samples [29]. To detect dissimilarity between samples, we calculated measures of beta diversity (weighted unifrac) on relative rather than absolute taxon abundance, and used permutational multivariate analysis of variance (PERMANOVA) as implemented in the *vegan* package [30] to test the hypothesis that location within a facility is a significant predictor of microbial community structure, while simultaneously adjusting for facility. Differences in microbial community composition across location was subsequently performed at the phylum level; we performed analysis of variance on the top three phyla identified across samples (Firmicutes, Proteobacteria, Cyanobacteria), adjusting for multiple testing with a Bonferroni correction. In order to identify taxa that are differentially abundant between locations within a facility, we first performed a variance stabilizing transformation as implemented in *DESeq2* [31], followed by hierarchical multiple testing as implemented in the *structSSI* package [32], an approach that takes advantage of the inherent structure present in microbial composition data. This allowed us to identify the differential abundance of microbes with greater power.

To determine the extent to which endotoxin can serve as a proxy for 16S rDNA copy number or the richness and evenness of microbial communities as determined by 16S rDNA sequencing, Spearman correlation was calculated between these measures.

In order to determine the proportion of a worker’s microbiome that is attributable to his work environment, we used SourceTracker [33], a Bayesian community-wide microbial source tracking algorithm, to estimate the proportion of the oral, nasal, and skin microbiome attributed to the work microbiome of each participant before and after a standard 8-hour work shift in the dirty location of the cage wash area. “Sink” samples were considered to be oral, nasal, and skin samples collected from the 10 participants before and after their work shift; “Source” samples were air samples from personal air samplers worn by each participant during a standard 8-hour work shift. The analysis was stratified by pre- and post-shift samples.

Plotting was performed with the *ggplot2* [34] package. All analyses were performed in R 3.3.3. Two-sided p values of <0.05 were considered significant.

## Results

Sequencing was successfully performed on all environmental and human samples (see **S2 Fig** for sequencing depth by sample type and rarefaction curves). The field blank showed negligible contamination (see Table in **S2 Table**).

For our first aim evaluating the occupational microbial environment in these mouse facilities, we analyzed sixteen paired samples from four locations within four facilities. Average endotoxin levels were low (**Table 1**), with median levels ranging from undetectable to 1.0 EU/m^3^, with no statistically significant differences between locations (p = 0.28) although there was a trend towards higher endotoxin levels in the dirty cage wash areas. There were significant differences in 16S rDNA gene copy number between locations (p = 0.04), with the highest levels detected in the dirty cage wash area. Mouse allergen levels were also highest in the dirty cage wash area (5.6 [4.8–11.2] ng/m^3^), with significant differences between location (p = 0.01). While we observed differences in these exposures between locations, there were no statistically significant differences in each of these exposures when comparing between facilities.

**Table 1:** Summary measures of endotoxin, mouse allergen, and 16S rDNA copies per cubic meter of air sampled by location (animal, dirty, middle, set up locations). Values depicted are median [interquartile range]. Average endotoxin levels were low and not statistically different between locations, although measures of total bacterial count with quantitative PCR of 16S rDNA showed significant differences in bacterial counts between locations, with the highest counts in the dirty location. Spearman correlation between endotoxin and 16S rDNA copies was 0.62. Mouse allergen levels were also significantly different across location.

Measure	Location				p-value
	Animal Room	Dirty (Area)	Middle	Set Up	
Endotoxin, EU/m^3^	0 [0–0.2]	1.0 [0.8–1.2]	0.7 [0.4–1.1]	0.5 [0.2–1.0]	0.28
16S rDNA, copies/m^3^	2,598.7 [1951.9–4842.0]	28,396.0 [25374.7–30853.6]	4,256.1 [2943.3–5064.7]	4,031.2 [2068.1–5531.2]	<0.001
Mouse allergen, ng/m^3^	1.2 [0.7–2.0]	5.6 [4.8–11.2]	0.3 [0.2–0.5]	0.3 [0.2–0.5]	0.01
Observations	4	4	4	4	

16S rDNA sequencing revealed significant differences in microbial diversity and community composition of microbes between locations, with distinct communities determined by location within facility rather than by facility. We identified significant differences in richness as measured by observed OTUs (p = 0.009 for location, p = 0.44 for facility) and Chao 1 (p = 0.006 for location, p = 0.46 for facility), although there were no statistically significant differences in α diversity measures that reflect both richness and evenness such as the Shannon (p = 0.39 for location, p = 0.76 for facility) and inverse Simpson (p = 0.29 for location, p = 0.60 for facility) indices. A sensitivity analysis using rarefied OTU tables did not change these results, with location being significantly associated with observed OTUs (p = 0.02) and Chao 1 (p = 0.01).

**Fig 1:**
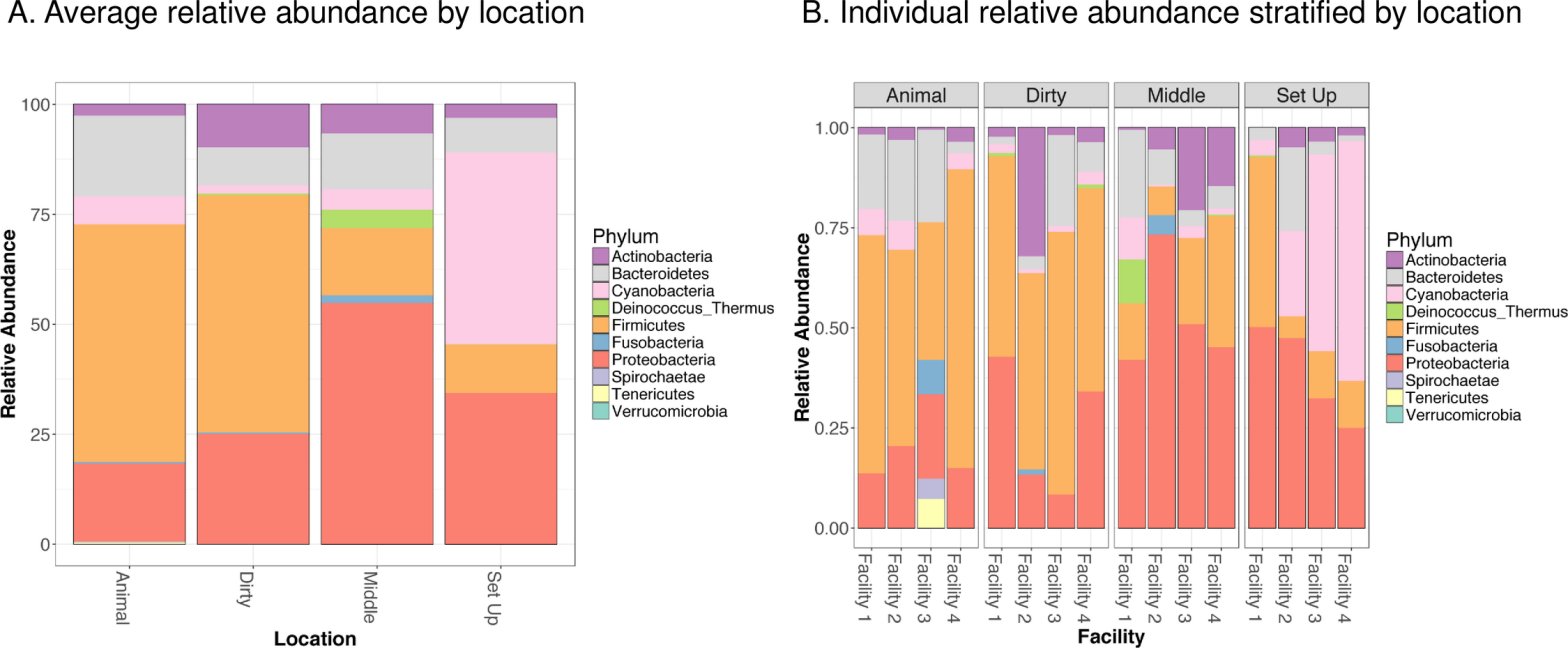
Microbial community composition measured by 16S rDNA sequencing shows significant differences between locations despite no differences in measured endotoxin levels. Relative abundance is plotted on the x axis, with each color representing a unique phylum, whereas location is plotted on the y axis. **Fig 1a** shows average relative abundance by location, **Fig 1b** shows relative abundance in each sample stratified by location. Although endotoxin levels were not significantly different between locations, statistically significant differences in community richness and relative abundance of *Firmicutes* (p = 0.006), *Proteobacteria* (p = 0.03), and *Cyanobacteria* (p = 0.04) were identified between locations.

**Fig 2:**
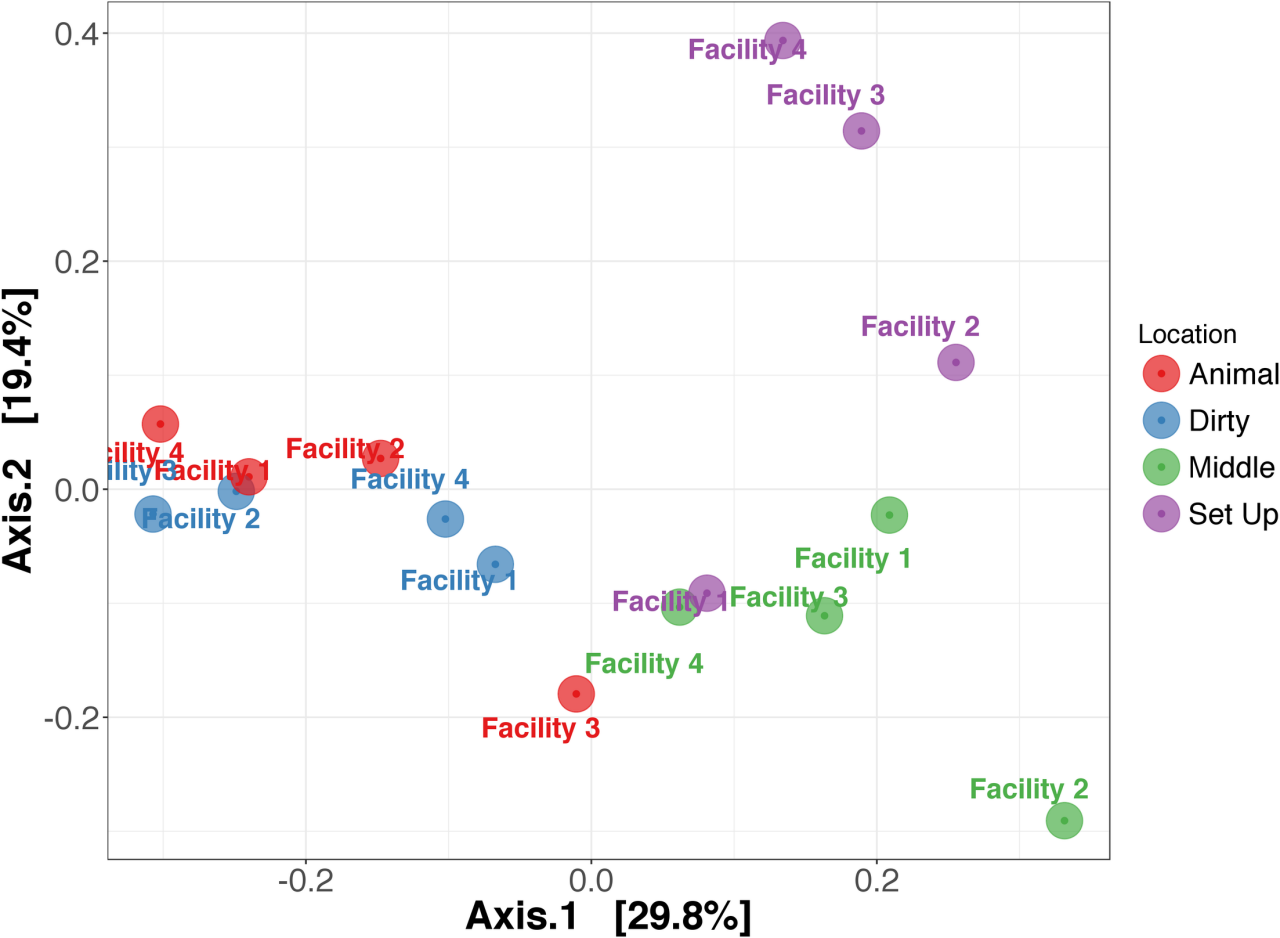
Principle coordinates analysis using weighted unifrac as the measure of dissimilarity shows that microbial community structure differs between locations in a facility rather than between different facilities. Microbial communities that are more similar will be closer together. Each color indicates a location within each facility (animal, dirty, middle, set up locations), while text represents each facility. Note that colors (indicating location) visually cluster together, not text (indicating facility). Statistical testing with permutational analysis of variance (PERMANOVA) indicates that location is a stronger predictor of microbial community structure (R^2^ = 0.41, p = 0.002) than facility (R^2^ = 0.14, p = 0.59).

When looking at microbial community composition (**Fig 1**), there were statistically significant differences in the relative abundance of Firmicutes (p = 0.006), Proteobacteria (p = 0.03), and Cyanobacteria (p = 0.04) between locations. The top taxa that were differentially abundant between locations are depicted in a Table in **S3 Table**. When evaluating the dissimilarity of microbial communities using PERMANOVA (**Fig 2**), location within a facility was a strong predictor of microbial community structure (i.e. animal vs. dirty vs. middle vs. setup locations, R^2^ = 0.41, p = 0.002), whereas facility was not associated with microbial community structure (i.e. facility 1 vs. facility 2 vs. facility 3 vs facility 4, R^2^ = 0.14, p = 0.59).

In order to determine the extent to which endotoxin levels can serve as a proxy for other measures of the microbial environment, we calculated the Spearman correlation between endotoxin levels and other microbial measures. We found a modest correlation between measured endotoxin and 16S rDNA copy number (Spearman ρ = 0.54, p = 0.09). Similarly, correlation between measured endotoxin levels and various measures of alpha diversity was modest as follows: Spearman ρ = 0.47, p = 0.14 for Observed OTUs; Spearman ρ = 0.46, p = 0.15 for the Chao 1 index; Spearman ρ = 0.67, p = 0.02 for the Shannon index; Spearman ρ = 0.59, p = 0.06 for the Inverse Simpson index. Notably, correlation between measured endotoxin and mouse allergen levels was low (Spearman ρ = 0.35, p = 0.30)

**Fig 3:**
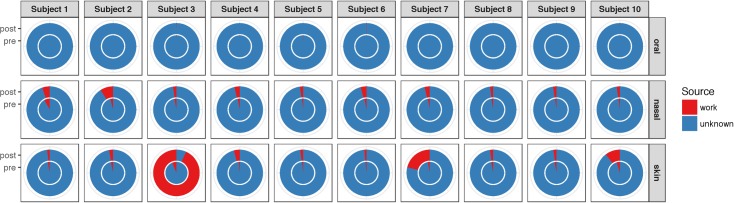
Proportion of human microbiome tracked to work environment as a source. Using SourceTracker [33], a Bayesian community-wide microbial source tracking algorithm, we estimated the proportion of the oral, nasal, and skin microbiome attributed to the work microbiome of each participant before (pre-shift, inner circle) and after (post-shift, outer circle) a standard 8-hour work shift in the dirty location of the cage wash area. 10 participants working in the dirty cage wash areas of the four facilities had human microbiome samples collected before and after their work shift; during their work shift, each wore personal air samplers to obtain measures of their work microbiome. For example, the proportion of subject 3’s skin microbiome attributed to his work microbiome increased from 6.2% (inner circle) to 93.2% (outer circle) after his work shift (column 3, row 3). Differences in the pre- and post-shift proportion of the oral, nasal, and skin microbiome did not reach statistical significance.

For our second aim, we evaluated whether the work microbiome of workers had an impact on the human microbiome of workers, and whether the human microbiome of exposed workers in the dirty cage wash areas changed during a standard 8-hour work shift (see **Table 2**for characteristics of workers). The work microbiome obtained from personal air sampling along with the nasal, oral, and skin microbiome of these ten workers is as depicted in **S3 Fig**. We used SourceTracker to identify the proportion of “contamination” in each worker’s oral, nasal, and skin microbiome attributed to the work microbiome of each participant. This was done for human samples collected both before and after a standard 8-hour work shift in the dirty location of the cage wash area (**Fig 3**). The average proportion of each worker’s *pre-shift* microbiome attributed to their work microbiome as a source was as follows: 0.1 ± 0.1% for the oral microbiome; 3.1 ± 1.9% for the nasal microbiome; 3.0 ± 1.5% for the skin microbiome. The average proportion of each worker’s *post-shift* microbiome attributed to their work microbiome as a source was as follows: 0 ± 0% for the oral microbiome; 3.7 ± 2.1% for the nasal microbiome; 14.1 ± 28.5% for the skin microbiome. The change in the proportion of the worker’s microbiome when comparing post- vs. pre- shift samples did not reach statistical significance (p = 0.14 for the oral microbiome, p = 0.41 for the nasal microbiome, and p = 0.23 for the skin microbiome).

**Table 2:** Characteristics of study participants.

Characteristic	Mean ± SD or N (%)
Participants	10
Age (years)	46.5 ± 11.9
Men	10 (100%)
Body mass index (kg/m^2^)	29.5 ± 7.6
Years worked	10.4 ± 2.2
Doctor diagnosis of asthma	3 (30%)
Doctor diagnosis of hayfever	5 (50%)
Lifetime nonsmoker	3 (30%)
Current smoker	4 (40%)
Pack-years	11.3 ± 3.6
FEV_1_ (liters)	2.8
% predicted FEV_1_	87.0 ± 15.3
FVC (liters)	3.6
% predicted FVC	85.6 ± 17.6
FEV_1_/FVC < 0.70	2 (20%)
+ Bronchodilator response	2 (20%)
Exhaled nitric oxide (ppb)	19.4 ± 14.6
Work-related symptoms	3 (30%)
Cross-shift change in FEV_1_ (mL) ^a^	40 ± 179.7
Cross-shift change in exhaled nitric oxide (ppb)	2.8 ± 5.3

^a^ Two subjects had pre- or post-shift spirometry that did not meet ATS criteria for reproducibility and so were excluded from the analysis

Of note, there were no significant differences in community structure of the environmental microbiome in the dirty cage wash areas as measured by area samplers vs. personal samplers worn by the participants (R^2^ = 0.05, p = 0.95, see **S4 Fig** for relative abundance of major phyla in area vs. personal samplers).

## Discussion

We have demonstrated that in this study, unsurprisingly, endotoxin is not a reliable proxy for measures of microbial load assessed with quantitative PCR, and endotoxin is not a reliable proxy for microbial diversity as measured by 16S amplicon sequencing. Microbial community structure in these facilities is predicted by location within each facility rather than by facility, and likely reflects the specialized functions of each location. A non-zero proportion of the worker’s nasal and skin microbiome in these facilities can be traced to the worker’s environment as a source; though there were fluctuations in this proportion after as compared to before a work shift, these changes did not reach statistical significance. Taken together, these data suggest that it may be important to incorporate microbial DNA sequencing in future studies on occupational microbial exposure, as measurement of endotoxin alone is not a proxy for overall microbial quantity or identity. Occupational microbial exposures appear to influence the human microbiome of workers, and further research is required to determine if this has implications for health effects independent of microbial toxin exposure.

To our knowledge, this is the first environmental study that has characterized the microbial environment of animal care facilities using 16S rDNA sequencing. Prior studies have identified high levels of endotoxin as well as mouse allergen in this work environment [35], and found a correlation between endotoxin exposure and respiratory symptoms that is independent of allergen exposure [35]. Although we report lower mouse allergen and endotoxin levels than prior studies, the major determinant of the environmental microbiome in the animal rooms and dirty cage wash area is likely the presence of mice. The mouse gut microbiome is well characterized [36] and the major phyla present in the mouse gut are also those we report in the animal rooms and dirty cage wash areas, suggesting that our results will likely generalize to other mouse facilities. The middle cage wash area had a striking presence of Deinococcus Thermus, bacteria that tend to be resistant to environmental extremes. This may be related to the use of heated water (up to 180 degrees Fahrenheit) to wash the cages as they pass from the dirty to middle cage wash areas. The setup area had the highest relative abundance of Cyanobacteria when compared across locations. Cyanobacteria obtain their energy through photosynthesis, and while it may be unexpected that these bacteria would be present when all the mouse facilities surveyed were located in basements, we suspect the source of the Cyanobacteria is from the woodchip bedding used for the mouse cages, as the setup area is where the cleaned cages are filled with litter and food before autoclaving. Because the function of each location within the mouse facility is different, it is not surprising that we found that differences in microbial structure between locations within a facility.

There are few studies evaluating the effect of animal-related work on the human microbiome, though none have simultaneously measured both the environmental microbiome as well as the human microbiome in these occupational studies. Respiratory colonization of livestock workers by strain-specific MRSA has been described [37, 38]. A stool microbiome study was performed in poultry abattoir workers where stool was serially collected during the peak season of *Campylobacter* infection in chickens in Sweden [14]. Of 31 workers, 7 acquired *Campylobacter*, and furthermore there were significant changes in the gut microbiome of all workers over a 5-month period, supporting the idea that work-related transmission of microbes to humans does occur. However, there was no control group in this study, making it difficult to disentangle seasonal effects from those due to occupational microbial exposures, and the environmental microbiome was not assessed, making it difficult to pinpoint the reason for the observed changes in the gut microbiome.

Surprisingly, few studies have directly compared endotoxin against qPCR of 16S rDNA copy number and 16S rDNA sequencing. Traditionally, occupational microbial exposures have most frequently been approximated with measurement of endotoxin, which is present only in gram-negative bacteria [39]. Some investigators have assumed that high endotoxin levels can serve as a proxy for high total microbial exposures, or exposure to high microbial diversity [40]. Our study does not support this assumption. There is one study in settled dust from homes that has compared bacterial load using qPCR against measurement of endotoxin and found at best a moderate correlation between qPCR and endotoxin [41]; this is consistent with our findings. A recent study comparing endotoxin, 16S qPCR, and 16S rDNA sequencing in settled dust found inconsistent associations between these measures with asthma severity [6]. A low diversity of microbial exposure as measured by 16S rDNA sequencing was associated with decreased asthma symptoms, whereas bacterial concentration as measured by 16S qPCR was not associated with symptoms, and endotoxin exposure was associated with a trend towards increased asthma symptoms. This highlights the observation that each of these measures likely reflect a different aspect of microbial exposure, and are not interchangeable.

There are mechanistic reasons for why it may be relevant for human health to identify microbes in the environment using sequencing rather than only measuring microbial toxin quantity. The measurement of microbial exposures in most epidemiologic studies have focused on the measurement of endotoxin. The most widely used assay for endotoxin is the Limulus amebocyte lysate assay [42], an assay based on the principal that endotoxin initiates an enzymatic clotting cascade in blood of Atlantic horseshoe crabs. While a sensitive assay for the detection of endotoxin, it is unclear that potency measured in this way translates to human responses [43]. A recent publication demonstrated that endotoxin from different bacterial species elicits opposing responses in humans. Endotoxin from *Escheria coli* elicited a robust cytokine response in human peripheral blood mononuclear cells, while endotoxin from *Bacteroides dorei* inhibited the ability of *Escheria coli* endotoxin to stimulate a cytokine response [44]. This example highlights the importance of determining microbial identity rather than just measuring toxin levels when determining associations with human health.

A strength of our study is the direct comparison of three measures of microbial exposure in an occupational environment, as well as the simultaneous measure of the environmental and human microbiome. We did not have evidence of high abundance contamination, a common problem in microbiome studies [45]. The use of each worker *as their own control* is a strength; repeated measures of workers over a short period of time (an 8-hour work shift) limits confounding by seasonal factors, although we did not control for variability from short term environmental influences such as food intake that occurred during this time period. For both human and environmental samples, we used methods for sample collection, DNA extraction, sequencing, and initial data processing that reflects methods used in the Human Microbiome Project [21] and Earth Microbiome Project [46] allowing comparability with other studies. Our study has a few limitations. Our sample size was small, which limits our power to detect differences between environmental samples and between pre- and post-shift human samples. Because of our small sample size, this study was not designed to link measures of microbial identity or microbial community structure to health effects. Although we used each worker as their own control to detect changes in their microbiome over the course of a work shift, we did not have an unexposed control group to define what is normal variation in the human microbiome. However, our finding that a proportion of the source of even the pre-shift skin and nasal microbiome of workers can be traced to their work environment offers preliminary evidence for the concept that the adult human microbiome can be impacted by microbes in their work environment. Perhaps the major limitation of our study is that due to the short time period over which workers were tested, we do not know how durable the changes in the human microbiome from work exposures are. Our study design using prevalent hire workers tested over a work shift was due to feasibility; on average, less than one new worker is hired annually within these four facilities, and most new hires have previously worked in animal care, preventing us from using newly hired workers to study the long-term effects of microbial exposure from animal care work. Despite these limitations, we do demonstrate that a proportion of each worker’s pre-shift skin and nasal microbiome can be traced to their work environment as a source, and show that endotoxin cannot serve as a proxy for microbial DNA qPCR and sequencing. A prior randomized experimental study in which healthy adults ingested a specific strain of *Lactobacillus* for one week found persistent increases in the stool of this strain three weeks after the last exposure [47]. This suggests that it is possible that introduction of new environmental microbes can have persistent effects on a healthy adult human microbiome. We hope to address these shortcomings in future work, but our study raises important questions about how work-related microbial exposures may alter colonization patterns of the human microbiome.

In summary, we demonstrate that the microbial structure of academic animal care facilities varies significantly by location rather than by individual facility; these differences are not well approximated with measurement of endotoxin levels, limiting our ability to extrapolate from prior occupational studies relying only on endotoxin for exposure assessment. We find evidence that a non-zero proportion of the nasal and skin microbiome of workers can be traced to their work environment as a source. This opens up the possibility that work-related microbes may impact health through a mechanism other than microbial toxin exposure. Further research on the long-term stability of changes in the adult human microbiome due to occupational microbial exposure, the factors that impact susceptibility to colonization by microbes in the work environment, and the health effects of the workplace microbiome, should be considered. This approach has the potential to change how we set safe microbial work exposure standards in the future.

## Supporting information

S1 Fig Representative pictures of each location within an animal care facility. A typical animal room and dirty, middle, and set up areas of cage wash are depicted. Animal rooms are where the mice are housed. Within the cage wash areas, the dirty location is where litter and food pellets of used cages are dumped, and cages subsequently placed in an industrial washer. In the middle location, washed cages are set in racks to dry. In the setup location, dried cages are filled with clean litter and food.
(DOCX)

S2 Fig **Rarefaction curves (a) and number of reads by sample type (b).** Color of bars indicates sample type.
(DOCX)

S3 Fig Microbiome of work environment obtained from personal air sampling as well as the oral, nasal, and skin microbiome of 10 workers in the dirty location of the four animal care facilities. Relative abundance depicted at the phylum level.
(DOCX)

S4 Fig Microbial community composition comparing area vs. personal samplers in dirty cage wash area. Relative abundance depicted at the phylum level. Statistical testing with permutational analysis of variance (PERMANOVA) showed no significant differences in community composition of the environmental microbiome in the dirty cage wash areas as measured by area samplers vs. personal samplers worn by the participants (R^2^ = 0.05, p = 0.95).
(DOCX)

S1 Table Accession numbers for raw sequence data deposited in the NCBI read sequence archive. (XLSX)

S2 Table Results from field blank. A pair of field blank samples using filters from the same batch as actual samples, but was not connected to a sampling pump, underwent endotoxin analysis and 16S sequencing. Endotoxin levels were below the limit of detection in the field blank. 17 reads were identified on 16S sequencing.
(DOCX)

S3 Table OTUs that are differentially abundant in the environmental microbiome between locations of animal care facilities. Hierarchical multiple testing was performed on variance stabilized data from area air samples to identify taxa that were differentially abundant across locations within a facility, adjusting for multiple testing.
(DOCX)
