# Supplementary material for: Impact of environmental microbiota on human microbiota of workers in academic mouse research facilities: An observational study
Source: PLoS One. 2017 Jul 13;12(7):e0180969. doi: 10.1371/journal.pone.0180969 (PMC5509249; doi:10.1371/journal.pone.0180969)

**S1 Fig**. Representative pictures of each location within an animal care facility. A typical animal room and dirty, middle, and set up areas of cage wash are depicted. Animal rooms are where the mice are housed. Within the cage wash areas, the dirty location is where litter and food pellets of used cages are dumped, and cages subsequently placed in an industrial washer. In the middle location, washed cages are set in racks to dry. In the setup location, dried cages are filled with clean litter and food.


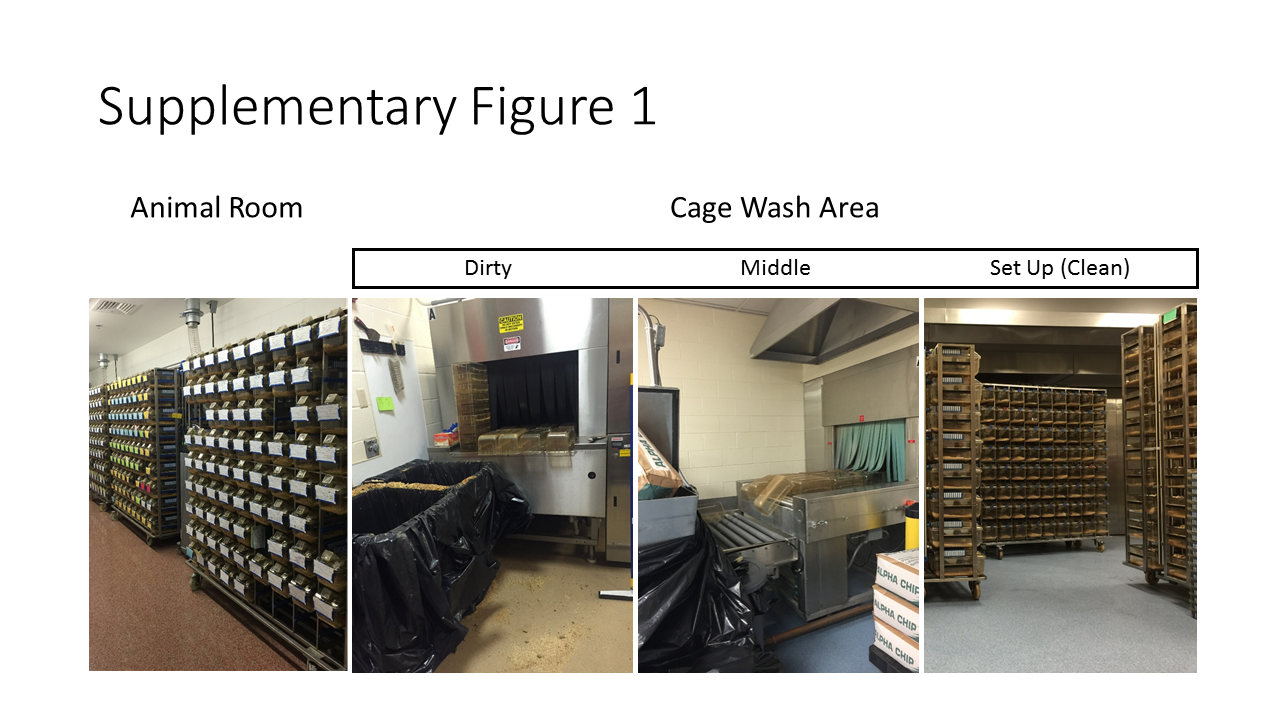

Supplement: S1 Fig — A typical animal room and dirty, middle, and set up areas of cage wash are depicted. Animal rooms are where the mice are housed. Within the cage wash areas, the dirty location is where litter and food pellets of used cages are dumped, and cages subsequently placed in an industrial washer. In the middle location, washed cages are set in racks to dry. In the setup location, dried cages are filled with clean litter and food. (DOCX) [file pone.0180969.s001.docx]
