# Supplementary material for: Impact of environmental microbiota on human microbiota of workers in academic mouse research facilities: An observational study
Source: PLoS One. 2017 Jul 13;12(7):e0180969. doi: 10.1371/journal.pone.0180969 (PMC5509249; doi:10.1371/journal.pone.0180969)
Supplement: S2 Fig — Rarefaction curves (a) and number of reads by sample type (b). Color of bars indicates sample type. (DOCX) [file pone.0180969.s002.docx]

**S2 Fig. Rarefaction curves (a) and number of reads by sample type (b).** Color of bars indicates sample type.

|  |
| --- |
