# Supplementary material for: Impact of environmental microbiota on human microbiota of workers in academic mouse research facilities: An observational study
Source: PLoS One. 2017 Jul 13;12(7):e0180969. doi: 10.1371/journal.pone.0180969 (PMC5509249; doi:10.1371/journal.pone.0180969)
Supplement: S3 Fig — Relative abundance depicted at the phylum level. (DOCX) [file pone.0180969.s003.docx]

**S3 Fig. Microbiome of work environment obtained from personal air sampling as well as the oral, nasal, and skin microbiome of 10 workers in the dirty location of the four animal care facilities.** Relative abundance depicted at the phylum level.

**
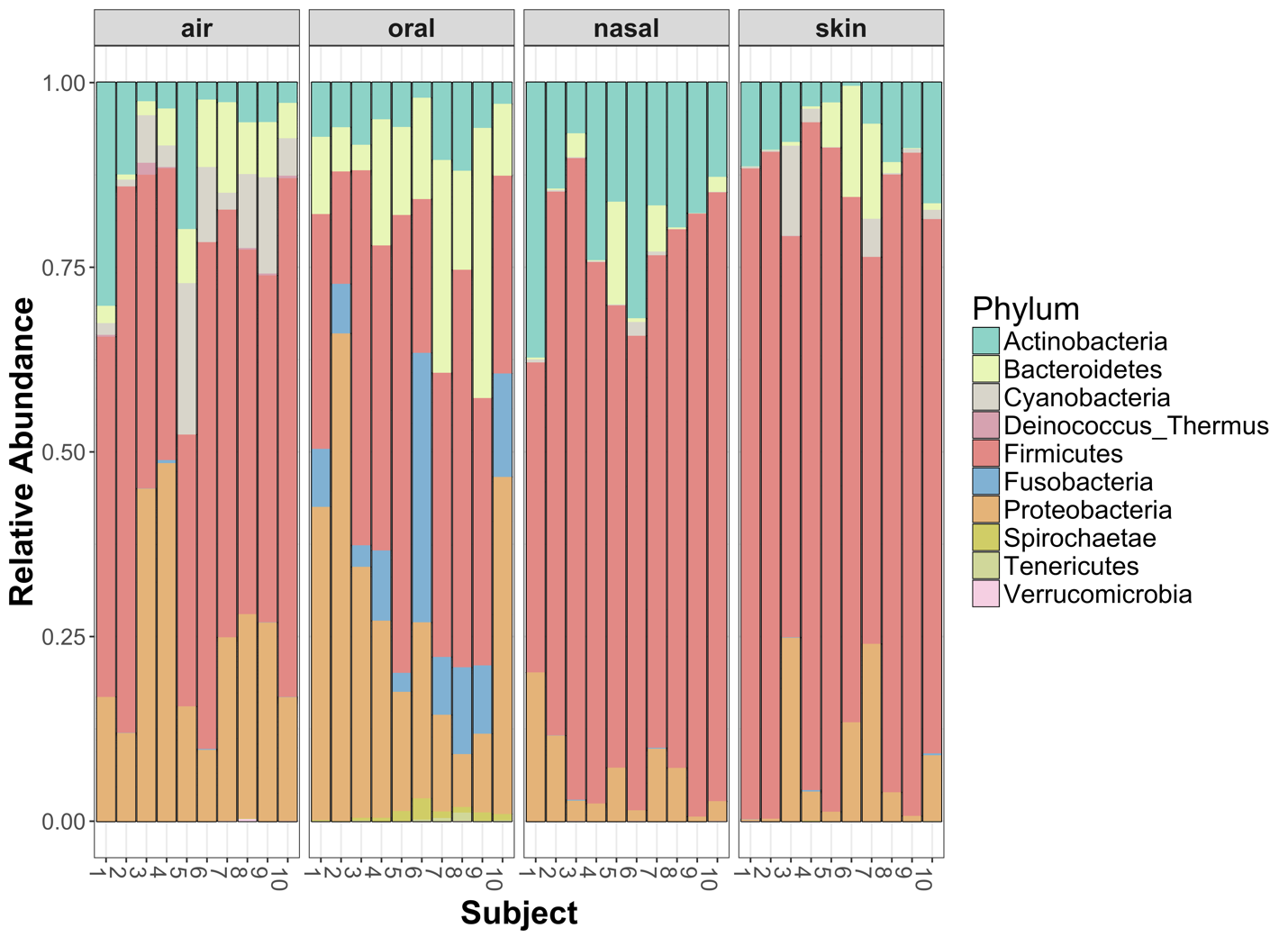
**
