# Supplementary material for: Impact of environmental microbiota on human microbiota of workers in academic mouse research facilities: An observational study
Source: PLoS One. 2017 Jul 13;12(7):e0180969. doi: 10.1371/journal.pone.0180969 (PMC5509249; doi:10.1371/journal.pone.0180969)
Supplement: S4 Fig — Relative abundance depicted at the phylum level. Statistical testing with permutational analysis of variance (PERMANOVA) showed no significant differences in community composition of the environmental microbiome in the dirty cage wash areas as measured by area samplers vs. personal samplers worn by the participants (R2 = 0.05, p = 0.95). (DOCX) [file pone.0180969.s004.docx]

**S4 Fig**. **Microbial community composition comparing area vs. personal samplers in dirty cage wash area.** Relative abundance depicted at the phylum level. Statistical testing with permutational analysis of variance (PERMANOVA) showed no significant differences in community composition of the environmental microbiome in the dirty cage wash areas as measured by area samplers vs. personal samplers worn by the participants (R^2^ = 0.05, p = 0.95).

**
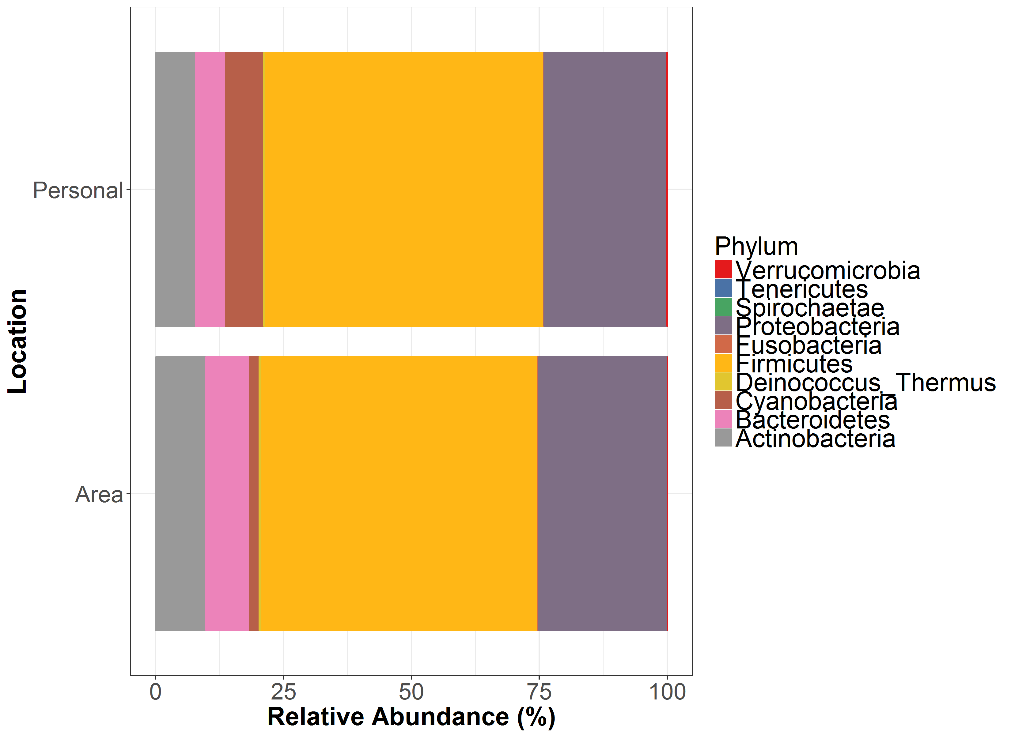
**
