# Supplementary material for: Impact of environmental microbiota on human microbiota of workers in academic mouse research facilities: An observational study
Source: PLoS One. 2017 Jul 13;12(7):e0180969. doi: 10.1371/journal.pone.0180969 (PMC5509249; doi:10.1371/journal.pone.0180969)
Supplement: S2 Table — A pair of field blank samples using filters from the same batch as actual samples, but was not connected to a sampling pump, underwent endotoxin analysis and 16S sequencing. Endotoxin levels were below the limit of detection in the field blank. 17 reads were identified on 16S sequencing. (DOCX) [file pone.0180969.s006.docx]

**S2 Table. Results from field blank**

A pair of field blank samples using filters from the same batch as actual samples, but was not connected to a sampling pump, underwent endotoxin analysis and 16S sequencing. Endotoxin levels were below the limit of detection in the field blank. 17 reads were identified on 16S sequencing.

The 17 annotated reads identified in the field blank belonged to the 5 OTUs detailed below. The most abundant OTU with 7 reads is from the *Variovorax* genus, which has been listed in prior publications as being found in the microbiome of extraction kits (i.e. the “kitome”)[1]. Note that even the most rigorously performed amplicon sequencing studies will have taxa sequenced from negative controls due to contaminants in the extraction kits [1] or from barcode bleed [2]. When 16 laboratories (many considered leaders in the microbiome field) participated in the Microbiome Quality Control project (MBQC), typically *several hundred putative OTUs* were obtained from sequenced negative controls [2]. In our study, identifying 17 annotated reads would not be consistent with high abundant contamination.

| **OTU ID**  **(Silva)** | **# reads** | **Kingdom** | **Phylum** | **Class** | **Order** | **Family** | **Genus** |
| --- | --- | --- | --- | --- | --- | --- | --- |
| VrvSpe79 | 7 | Bacteria | Proteobacteria | Betaproteobacteria | Burkholderiales | Comamonadaceae | Variovorax |
| Unc69015 | 3 | Bacteria | Firmicutes | Clostridia | Clostridiales | Lachnospiraceae | Lachnospiraceae_ NK4A136_group |
| Unc00gqv | 3 | Bacteria | Firmicutes | Bacilli | Lactobacillales | Streptococcaceae | Streptococcus |
| UncO4293 | 3 | Bacteria | Firmicutes | Bacilli | Bacillales | Staphylococcaceae | Staphylococcus |
| ShcSp185 | 1 | Bacteria | Cyanobacteria | Chloroplast | o | f | g |

**References**

1. Salter SJ, Cox MJ, Turek EM, Calus ST, Cookson WO, Moffatt MF, et al. Reagent and laboratory contamination can critically impact sequence-based microbiome analyses. BMC biology. 2014;12:87. doi: 10.1186/s12915-014-0087-z. PubMed PMID: 25387460; PubMed Central PMCID: PMC4228153.

2. Sinha R, Abnet CC, White O, Knight R, Huttenhower C. The microbiome quality control project: baseline study design and future directions. Genome biology. 2015;16:276. doi: 10.1186/s13059-015-0841-8. PubMed PMID: 26653756; PubMed Central PMCID: PMCPMC4674991.
