# Supplementary material for: Impact of environmental microbiota on human microbiota of workers in academic mouse research facilities: An observational study
Source: PLoS One. 2017 Jul 13;12(7):e0180969. doi: 10.1371/journal.pone.0180969 (PMC5509249; doi:10.1371/journal.pone.0180969)
Supplement: S3 Table — Hierarchical multiple testing was performed on variance stabilized data from area air samples to identify taxa that were differentially abundant across locations within a facility, adjusting for multiple testing. (DOCX) [file pone.0180969.s007.docx]

**S3 Table. OTUs that are differentially abundant in the environmental microbiome between locations of animal care facilities.**

Hierarchical multiple testing was performed on variance stabilized data from area air samples to identify taxa that were differentially abundant across locations within a facility, adjusting for multiple testing.

| **Phylum** | **Family** | **Genus** | **OTU ID**  **(Silva)** | **Unadjusted p-value** | **Adjusted**  **p-value** |
| --- | --- | --- | --- | --- | --- |
| Proteobacteria | Enterobacteriaceae | Rahnella | Unc02h9n | 5.08E-09 | 1.02E-08 |
| Firmicutes | Lachnospiraceae | Lachnospiraceae_NK4A136_group | Unc69015 | 5.89E-09 | 1.18E-08 |
| Firmicutes | Staphylococcaceae | Staphylococcus | ShcSp185 | 0.001385907 | 0.002771815 |
| Proteobacteria | Rhodocyclaceae | Azospira | Unc02nyt | 0.002400168 | 0.004800336 |
| Bacteroidetes | Bacteroidales_S24_7_group | g | Unc03k14 | 0.002424496 | 0.004848991 |
| Firmicutes | Streptococcaceae | Lactococcus | LtcLa173 | 0.002573523 | 0.005147047 |
| Proteobacteria | Caulobacteraceae | Brevundimonas | BvdSp221 | 0.002847459 | 0.005694918 |
| Firmicutes | Lachnospiraceae | Lachnospiraceae_NK4A136_group | Unc03l1t | 0.007108085 | 0.007108085 |
| Cyanobacteria | f | g | UncO4293 | 0.010222562 | 0.020445124 |
| Proteobacteria | Rhodobacteraceae | Paracocccus | Unc01ixs | 0.011234361 | 0.022468722 |
| Proteobacteria | Neisseriaceae | uncultured | Unc048an | 0.034590357 | 0.034590357 |
| Proteobacteria | Neisseriaceae | uncultured | Unc0266w | 0.034590357 | 0.034590357 |
| Fusobacteria | Leptotrichiaceae | Leptotrichia | LprSpec3 | 0.034590357 | 0.034590357 |
| Fusobacteria | Leptotrichiaceae | Leptotrichia | LprSpec9 | 0.034590357 | 0.034590357 |
| Spirochaetae | Spirochaetaceae | Treponema_2 | UncTr374 | 0.034590357 | 0.034590357 |
| Spirochaetae | Spirochaetaceae | Treponema_2 | UncTr598 | 0.034590357 | 0.034590357 |
| Spirochaetae | Spirochaetaceae | Treponema_2 | UncTr406 | 0.034590357 | 0.034590357 |
| Spirochaetae | Spirochaetaceae | Treponema_2 | TreLeci3 | 0.034590357 | 0.034590357 |
| Spirochaetae | Spirochaetaceae | Treponema_2 | TreSpe13 | 0.034590357 | 0.034590357 |
| Spirochaetae | Spirochaetaceae | Treponema_2 | TreDent5 | 0.034590357 | 0.034590357 |
| Spirochaetae | Spirochaetaceae | Treponema_2 | UncTr588 | 0.034590357 | 0.034590357 |
| Bacteroidetes | Prevotellaceae | Prevotella_6 | PreBerg2 | 0.034590357 | 0.034590357 |
| Bacteroidetes | Prevotellaceae | Prevotella_6 | Unc52764 | 0.034590357 | 0.034590357 |
| Bacteroidetes | Prevotellaceae | Prevotella_6 | UncPrev6 | 0.034590357 | 0.034590357 |
| Bacteroidetes | Prevotellaceae | Prevotella_7 | Unc24653 | 0.034590357 | 0.034590357 |
| Bacteroidetes | Prevotellaceae | Prevotella_7 | PreEnoe3 | 0.034590357 | 0.034590357 |
| Bacteroidetes | Prevotellaceae | Prevotella_7 | UncPre51 | 0.034590357 | 0.034590357 |
| Bacteroidetes | Prevotellaceae | Prevotella_7 | PreBaro2 | 0.034590357 | 0.034590357 |
| Bacteroidetes | Prevotellaceae | Prevotella_7 | PreDen19 | 0.034590357 | 0.034590357 |
| Bacteroidetes | Prevotellaceae | Prevotella_7 | Unc27391 | 0.034590357 | 0.034590357 |
| Bacteroidetes | Prevotellaceae | Prevotella_7 | UncPre78 | 0.034590357 | 0.034590357 |
| Bacteroidetes | Porphyromonadaceae | Porphyromonas | Unc56026 | 0.034590357 | 0.034590357 |
| Bacteroidetes | Porphyromonadaceae | Porphyromonas | Unc02805 | 0.034590357 | 0.034590357 |
| Bacteroidetes | Porphyromonadaceae | Porphyromonas | UniEub50 | 0.034590357 | 0.034590357 |
| Bacteroidetes | Porphyromonadaceae | Porphyromonas | PpmUeno2 | 0.034590357 | 0.034590357 |
| Firmicutes | Lachnospiraceae | Oribacterium | Unid1487 | 0.034590357 | 0.034590357 |
| Firmicutes | Lachnospiraceae | Oribacterium | Unc013qp | 0.034590357 | 0.034590357 |
| Firmicutes | Lachnospiraceae | Lachnoanaerobaculum | Unc26321 | 0.034590357 | 0.034590357 |
| Firmicutes | Lachnospiraceae | Johnsonella | Unc96902 | 0.034590357 | 0.034590357 |
| Firmicutes | Lachnospiraceae | Johnsonella | Unc000pl | 0.034590357 | 0.034590357 |
| Firmicutes | Lachnospiraceae | Stomatobaculum | Unc006uq | 0.034590357 | 0.034590357 |
| Firmicutes | Lachnospiraceae | Stomatobaculum | LhnGenom | 0.034590357 | 0.034590357 |
| Firmicutes | Erysipelotrichaceae | Solobacterium | Unc037lz | 0.034590357 | 0.034590357 |
| Firmicutes | Erysipelotrichaceae | Bulleidia | BulExtr2 | 0.034590357 | 0.034590357 |
| Firmicutes | Veillonellaceae | Anaeroglobus | UncOr431 | 0.034590357 | 0.034590357 |
| Firmicutes | Veillonellaceae | Anaeroglobus | Unc02rhj | 0.034590357 | 0.034590357 |
| Actinobacteria | Bifidobacteriaceae | Alloscardovia | AsdOmnic | 0.034590357 | 0.034590357 |
| Actinobacteria | Bifidobacteriaceae | Scardovia | BifSpe27 | 0.034590357 | 0.034590357 |
| Actinobacteria | Actinomycetaceae | Actinomyces | AnmOriho | 0.034590357 | 0.034590357 |
| Actinobacteria | Actinomycetaceae | Actinomyces | Unc03why | 0.034590357 | 0.034590357 |
| Actinobacteria | Actinomycetaceae | uncultured | AnbSpeci | 0.034590357 | 0.034590357 |
| Actinobacteria | Actinomycetaceae | uncultured | Unid1078 | 0.034590357 | 0.034590357 |
| Actinobacteria | uncultured | g | UncAc223 | 0.034590357 | 0.034590357 |
| Actinobacteria | Geodermatophilaceae | Blastococcus | BlcAggr2 | 0.034590357 | 0.034590357 |
| Spirochaetae | Spirochaetaceae | Treponema_2 | TreSocr6 | 0.034590357 | 0.034590357 |
| Bacteroidetes | Prevotellaceae | Prevotella_7 | Unc17528 | 0.034590357 | 0.034590357 |
| Firmicutes | Lachnospiraceae | uncultured | Unc006vd | 0.034590357 | 0.034590357 |
| Actinobacteria | Microbacteriaceae | Curtobacterium | CurSpe68 | 0.017982598 | 0.035965196 |
| Bacteroidetes | Prevotellaceae | Prevotella_1 | Unid1506 | 0.034590357 | 0.037127757 |
| Proteobacteria | Sphingomonadaceae | Sphingomonas | Unc87107 | 0.020107585 | 0.04021517 |
| Proteobacteria | Pseudomonadaceae | Pseudomonas | Unc03oxy | 0.020457083 | 0.040914166 |
